# Bayesian structural equation modeling for post treatment health related quality of life among tuberculosis patients

**DOI:** 10.1371/journal.pone.0252205

**Published:** 2021-05-28

**Authors:** Mahalingam Vasantha, Malaisamy Muniyandi, Chinnaiyan Ponnuraja, Ramalingam Srinivasan, Perumal Venkatesan

**Affiliations:** 1 Department of Statistics, ICMR-National Institute for Research in Tuberculosis, Indian Council of Medical Research, Chetpet, Chennai, India; 2 Department of Health Economics, ICMR-National Institute for Research in Tuberculosis, Indian Council of Medical Research, Chetpet, Chennai, India; Tabriz University of Medical Sciences, ISLAMIC REPUBLIC OF IRAN

## Abstract

**Background:**

The use of Bayesian Structural Equation Model (BSEM) to evaluate the impact of TB on self-reported health related quality of life (HRQoL) of TB patients has been not studied.

**Objective:**

To identify the factors that contribute to the HRQoL of TB patients using BSEM.

**Methods:**

This is a latent variable modeling with Bayesian approach using secondary data. HRQoL data collected after one year from newly diagnosed 436 TB patients who were registered and successfully completed treatment at Government health facilities in Tiruvallur district, south India under the National TB Elimination Programme (NTEP) were used for this analysis. In this study, the four independent latent variables such as physical well–being (PW = PW1-7), mental well-being (MW = MW1-7), social well-being (SW = SW1-4) and habits were considered. The BSEM was constructed using Markov Chain Monte Carlo algorithm for identifying the factors that contribute to the HRQoL of TB patients who completed treatment.

**Results:**

Bayesian estimates were obtained using 46,300 observations after convergence and the standardized structural regression estimate of PW, MW, SW on HRQoL were 0.377 (p<0.001), 0.543 (p<0.001) and 0.208 (p<0.001) respectively. The latent variables PW, MW and SW were significantly associated with HRQoL of TB patients. The age was found to be significantly negatively associated with HRQoL of TB patients.

**Conclusions:**

The current study demonstrated the application of BSEM in evaluating HRQoL. This methodology may be used to study precise estimates of HRQoL of TB patients in different time points.

## Introduction

Tuberculosis (TB) disease causes significant negative impact on Health Related Quality of Life (HRQoL) of the patient [1]. The need for evaluation of TB therapeutic interventions is no longer restricted to clinical outcomes but also includes psycho-social well-being and HRQoL. The measurement of HRQoL are being used in direct patient care processes, clinical trials, program evaluations, and for monitoring health status in populations [2]. Quality of life has become an instrumental outcome measure in clinical research, and advances have been made in assessing the impact of many diseases on HRQoL.

HRQoL is a multidimensional concept which is evaluated by a number of different latent constructs such as physical function, general health, mental health and social relationships [3]. As these latent constructs often cannot be measured objectively and directly, they are treated as latent variables in HRQoL analysis. In medical statistics, traditional regression models are limited for modeling latent variables and may result in biased estimates.

The Bayesian SEM approach utilises prior information for updating the current information on the parameter and to estimate the parameters based on posterior distribution defined for latent variables [4–8]. There is paucity of studies which applied BSEM for modelling socio-psychological variables which are latent in nature [9–12]. With this background, we applied BSEM for understanding the structural relationship of the factors contributing to HRQoL of TB patients for the first time.

## Methodology

### Study design

This is a latent variable modeling with Bayesian approach using secondary data.

### Source of data

HRQoL data collected after one year from newly diagnosed 436 TB patients who were registered and successfully completed treatment (Table 1) at Government health facilities in Tiruvallur district, south India under the National TB Elimination Programme (NTEP) were used for this analysis [13]. The study was approved by the Institutional Ethics Committee of the National Institute for Research in Tuberculosis, Indian Council of Medical Research, Chennai.

**Table 1:** Distribution of TB patients’ well-being in different domains.

HRQoL domains	No.	%
**Physical well-being**		
Free from TB symptoms	263	60
Other health problem	96	22
Current general health**—**Absolutely normal	391	90
No body pain	300	69
Body pain interfere activity- Not at all	323	74
**Rate your health**		
Good	161	37
Very good	140	32
**Health status compared to pre-illness**		
Somewhat better	94	22
Much better	215	49
**Mental well-being**		
Happy most of the time	249	57
Never frustrate or impatient	234	54
Never feeling of fear or panic	220	51
Never depressed	124	28
Always free of tension	94	22
Always energetic	161	37
Feel low energy	192	44
**Social well-being**		
Visiting friends/relatives/neighbours	363	83
Feel free to attend social function	365	84
Discussing illness with family members	401	92
Feeling inhibited to discuss illness with friends	344	79

### Tool used to measure HRQoL

The 36 item Short Form Health Survey (SF-36) questionnaire was used to measure HRQoL of the TB patients [14]. All the components under each domain were given equal weight for calculating of quality of life scoring based on SF-36. All scales and the component scores were positively scored and higher scores represented better HRQoL.

### Model specification

**Fig 1:**
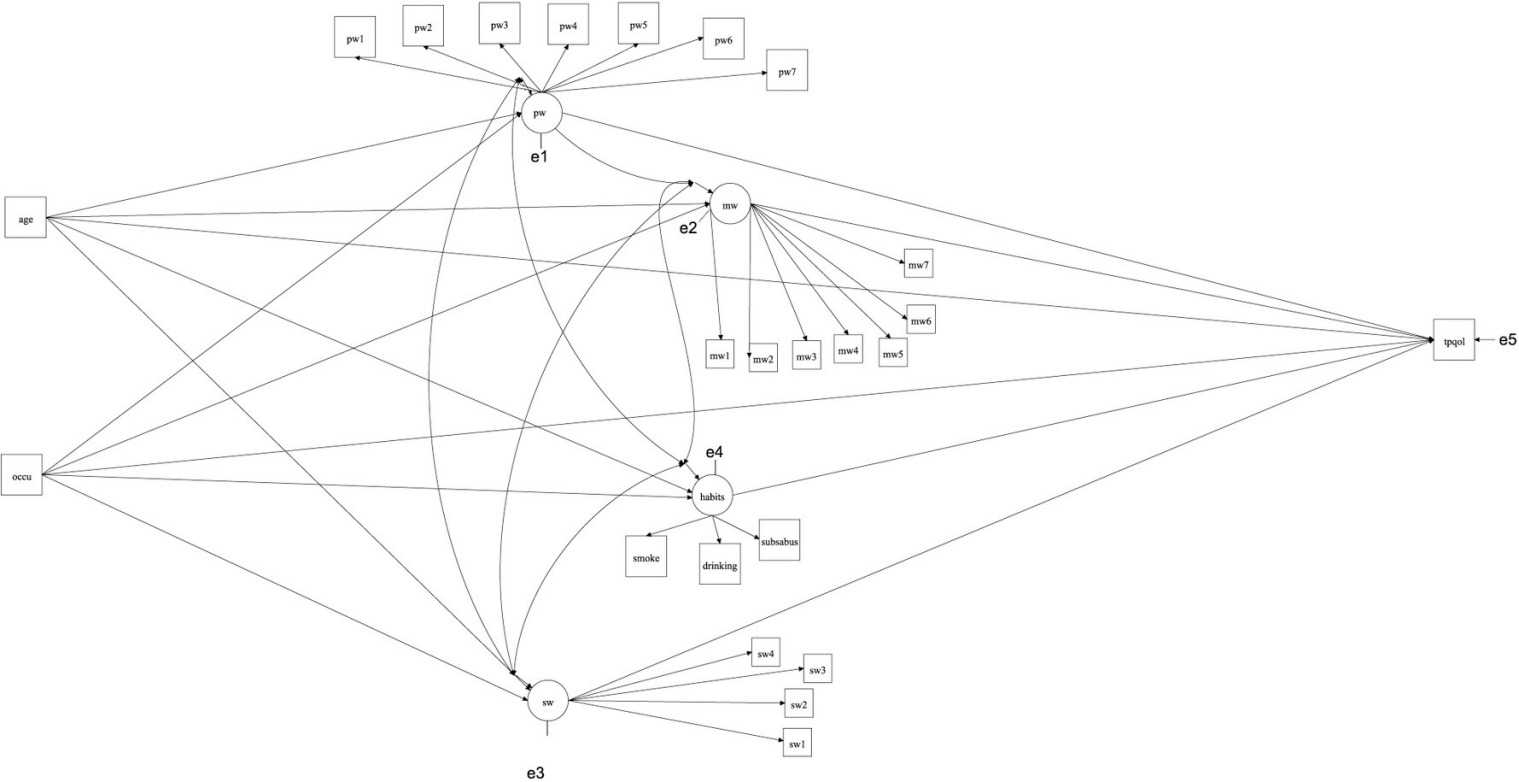
Path diagram for HRQoL of TB patients. Legend: pw- Physical well- being, mw-Mental well-being, sw-Social well-being, occu–Occupation, subsabus–substance abuse, smoke–smoking habits, tpqol–Health Related Quality of Life of tuberculosis patients.

In this study, 21 independent variables were used. The physical well-being variables were: Free from TB symptoms, other health problem, general health status, body pain, pain interfering normal activity, self-health rate and compared to pre- illness (PW1-PW7). The mental well-being variables were: happy person, frustrate/impatient, feeling fear/panic, depressed, free of tension, energetic and feeling low energy (MW1-MW-7). The social well-being variables were: visiting friends/relatives/neighbours, feel free to attend social functions, discussing illness with family members and feeling inhibited to discuss illness with friend (SW1-SW4). The life style related variables were: drinking alcohol, and smoking tobacco, substance abuse. The four independent latent variables which were grouped from these 21 independent variables are physical well–being (PW = PW1-7), mental well-being (MW = MW1-7) and social well-being (SW = SW1-4). The life style variables such as drinking alcohol, smoking tobacco, substance abuse were formed the latent variable habits. In addition, age and occupation variables were also considered for this model. The path diagram of different health domain influencing HRQoL of TB patients is given in Fig 1.

### BSEM analysis

The BSEM was constructed using Markov Chain Monte Carlo (MCMC) algorithm for identifying the factors that contribute to the HRQoL of TB patients who completed treatment. The M*Plus* software version 7.1 was used for analysis. The non-informative prior which is default in M*Plus* was used for BSEM. For BSEM, the model convergence was assessed by the trace plots and estimated potential scale reduction (EPSR). Convergence of the sequence has been achieved where the ESPR values are less than 1.2. A Posterior predictive p-value is used to test the goodness of fit of the posited model. The codes used for BSEM analysis are incorporated in S1 Appendix in S1 File.

## Results

### Estimates of Bayesian SEM

**Fig 2:**
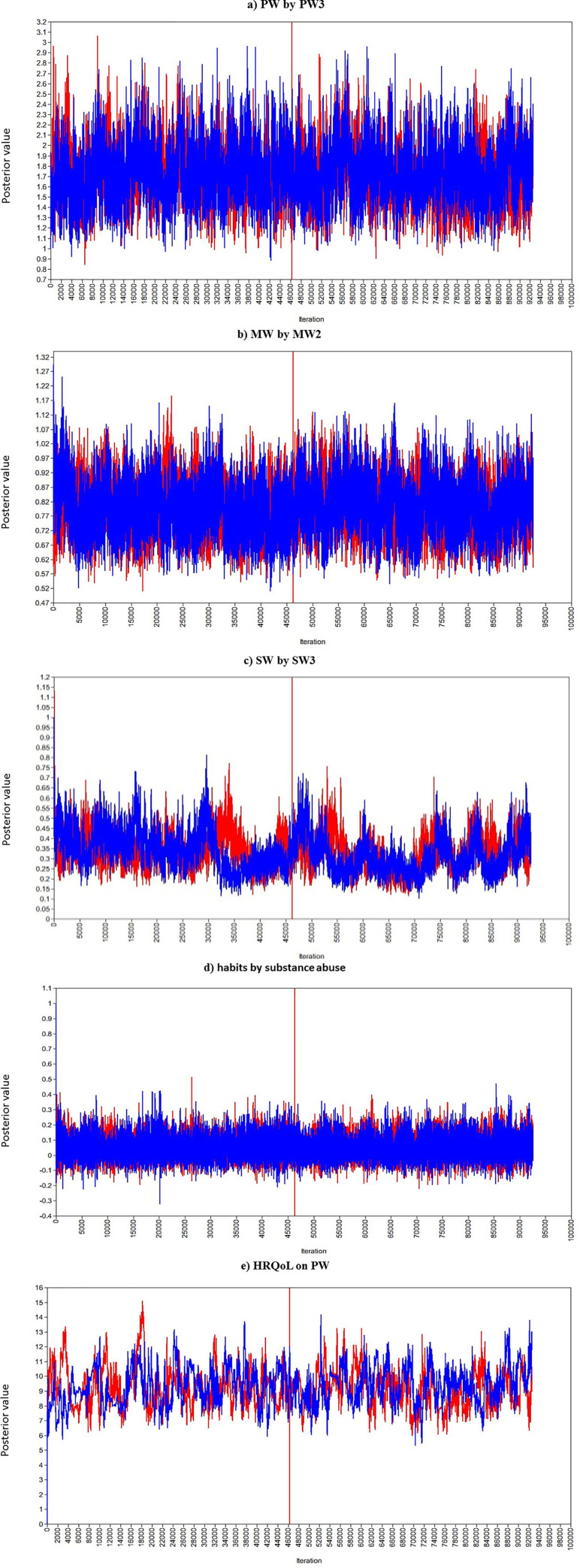
Trace plots of value of parameter for different iterations.

**Fig 3:**
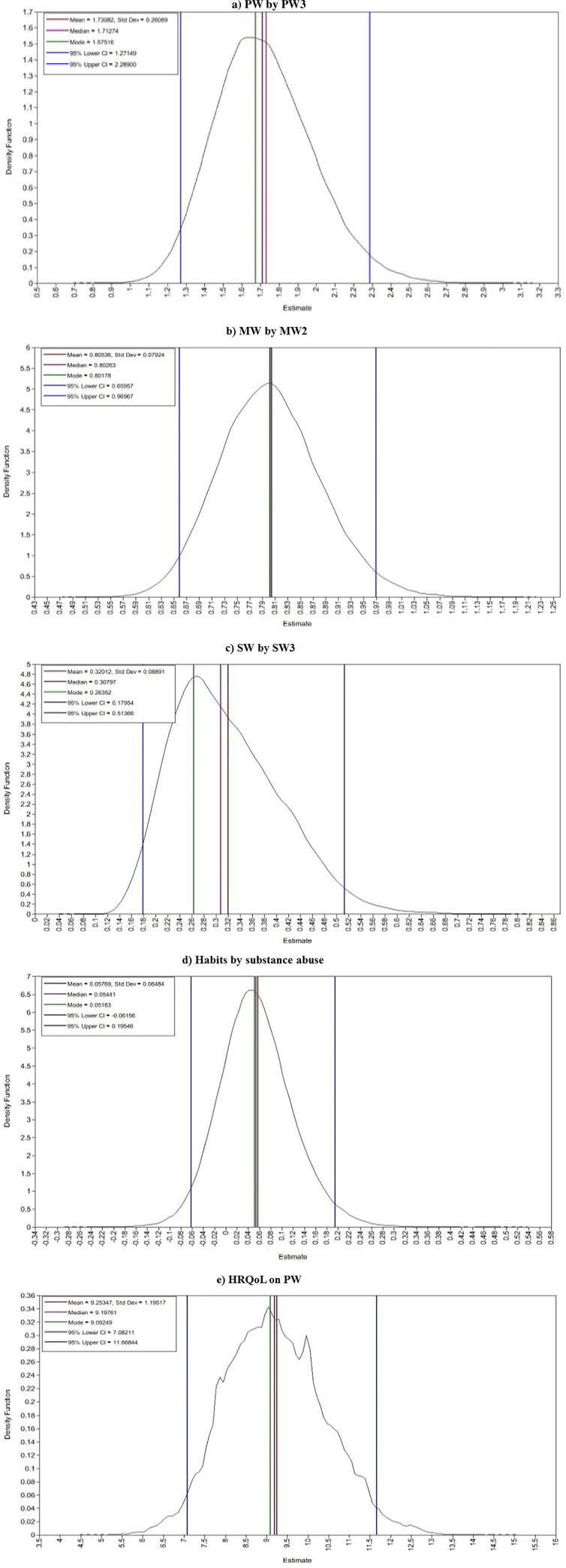
Posterior distribution of kernel density.

The Bayesian estimates for measurement model (Table 2) and structural model (Table 3) were obtained using 46,300 observations after discarding the first 46,300 burn-in iterations. It was observed that EPSR values are less than 1.2 after 39,000 iterations indicating model convergence. The trace plots for selected parameters demonstrates that a tight horizontal band which indicates the parameters are converged (Fig 2). The posterior probability density plots that indicate the posterior densities for these parameters are approximating normal density (Fig 3).

**Table 2:** Parameter estimates of measurement model for HRQoL of TB patients.

Factors	Estimated mean score	Posterior SD	95% credible interval	p-value
**Physical well-being by**				
Free from TB symptoms	0.586	0.038	(0.511, 0.657)	<0.001
Other health problem	0.408	0.058	(0.289, 0.515)	<0.001
General health status	0.778	0.039	(0.692, 0.844)	<0.001
Body pain	0.830	0.025	(0.777, 0.875)	<0.001
Pain interfering normal activity	0.864	0.024	(0.813, 0.906)	<0.001
Rate your health	0.838	0.020	(0.795, 0.873)	<0.001
Compared to pre-illness	0.661	0.034	(0.590, 0.723)	<0.001
**Mental well-being by**				
Happy person	0.786	0.023	(0.738, 0.828)	<0.001
Frustrate/impatient	0.714	0.028	(0.654, 0.765)	<0.001
Feeling fear/panic	0.717	0.029	(0.656, 0.769)	<0.001
Depressed	0.736	0.027	(0.679, 0.785)	<0.001
Free of tension	0.540	0.039	(0.461, 0.612)	<0.001
Energetic	0.814	0.021	(0.768, 0.851)	<0.001
Feeling low energy	0.763	0.025	(0.711, 0.808)	<0.001
**Social well-being by**				
Visiting friends/relatives/neighbours	0.961	0.019	(0.913, 0.984)	<0.001
Feel free to attend social functions	0.977	0.017	(0.934, 0.996)	<0.001
Discussing illness with family members	0.732	0.053	(0.617, 0.824)	<0.001
Feeling inhibited to discuss illness with friends	0.182	0.077	(0.028, 0.327)	<0.05
**Habits by**				
Smoking	0.866	0.041	(0.777, 0.931)	<0.001
Drinking	0.886	0.040	(0.796, 0.950)	<0.001
Substance abuse	0.096	0.103	(-0.108, 0.293)	0.179
**Physical well-being on**				
Age	-0.205	0.046	(-0.295, -0.112)	<0.001
Occupation	0.119	0.046	(0.025, 0.206)	<0.01
**Mental well-being on**				
Age	-0.161	0.044	(-0.244, -0.072)	<0.001
Occupation	0.115	0.043	(0.028, 0.196)	<0.01
**Social well-being on**				
Age	-0.141	0.064	(-0.268, -0.019)	<0.05
Occupation	0.249	0.058	(0.130, 0.354)	<0.001
**Habits on**				
Age	0.355	0.055	(0.243, 0.457)	<0.001
Occupation	0.244	0.059	(0.126, 0.356)	< 0.001

**Table 3:** Parameter estimates of structural model for HRQoL of TB patients.

Factors	Estimate	Posterior SD	95% credible interval	p-value
**HRQoL on**				
Physical Well-being	0.377	0.033	(0.312, 0.440)	<0.001
Mental Well-being	0.543	0.034	(0.478, 0.611)	<0.001
Social Well-being	0.208	0.026	(0.155, 0.259)	<0.001
Habits	-0.006	0.025	(-0.056, 0.040)	0.405
**HRQoL on**				
Age	0.040	0.019	(0.003, 0.076)	<0.05
Occupation	0.005	0.018	(-0.030, 0.040)	0.383
**Physical Well-being with**				
MW	0.752	0.032	(0.684, 0.810)	<0.001
SW	0.468	0.061	(0.341, 0.581)	<0.001
Habits	-0.121	0.073	(-0.263, 0.022)	<0.05
**Mental Well-being with**				
SW	0.622	0.050	(0.516, 0.711)	<0.001
Habits	0.088	0.071	(-0.051, 0.226)	0.108
**Social Well-being with Habits**	0.202	0.095	(0.009, 0.381)	<0.05

### Estimates of measurement model

It was observed that the estimate of all the variables except substance abuse were found to be significant with their respective latent variables (Table 2). The variables age and occupation were also found to be significant with the latent variables physical, mental, social well-being and habits.

### Estimates of structural model

The standardized estimate of physical, mental and social well-being on the variable percentage of total quality of life were 0.377 (p<0.001), 0.543 (p<0.001) and 0.208 (p<0.001) respectively. Hence mental health had the most important effect on HRQoL, followed in turn by physical health and social relationship. The physical, mental and social well-being latent variables were significantly associated with HRQoL, while bad habits was not significantly affecting the HRQoL negatively. The variables age was found to be significantly associated with HRQoL of TB patients. The covariance between social and physical, physical and mental, and social and mental well-being latent variables were found to be significant. Posterior predictive p-value for the model fit assessment was 0.045 which was reasonably close to the nominal 5% level.

## Discussion

Our study is first of its kind to use BSEM models to explore HRQoL data of newly diagnosed TB patients. Measurement of the HRQoL adds a new dimension to the evaluation of psychosocial variables of TB patients. We also examined the influence of covariates such as age and occupation on HRQoL scores for four physical, mental, social and life style latent variables. Of this, the mental well-being latent variable had most significant effect on HRQoL of TB patients followed by physical and social well-being latent variables.

The study findings highlights the significant association between mental well-being and chronic physical conditions of patients that significantly impact their HRQoL. Findings from a SEM modeling to detect HRQoL changes among cancer patients after invasive surgery found deteriorated physical well-being and improvement in mental well-being [15]. Other previous studies also reported almost similar findings [16–18].

In addition, we found that the covariate age was found to be significantly negatively associated with HRQoL of TB patients. This is logical as age increases, older persons have more health-related problems than the younger ages. This finding was corroborated with the findings from other studies [19–21]. To avoid this multiple effect, there is a need for an early diagnosis to improve the health of the elderly population in the later stages of life.

This study has few limitations; the first is we used the non-informative prior, which is default in the software M*Plus* that may influence our estimates. The second is that we used HRQoL of TB patients after completion of treatment and we didn’t get data during treatment.

## Conclusion

The current study demonstrated the application of BSEM in evaluating HRQoL. The physical, mental and social well-being latent variables were significantly associated with HRQoL of TB patients. This is a first attempt to develop methodology to apply BSEM to study HRQoL of TB patients. This methodology may be used to study precise estimates of HRQoL of TB patients in different time points and also to be applied to study the HRQoL of patients with other diseases.

## Supporting information

S1 File Mplus commands used for analyses. (DOCX)
