# Supplementary material for: Bayesian structural equation modeling for post treatment health related quality of life among tuberculosis patients
Source: PLoS One. 2021 May 28;16(5):e0252205. doi: 10.1371/journal.pone.0252205 (PMC8162623; doi:10.1371/journal.pone.0252205)
Supplement: S1 File — (DOCX) [file pone.0252205.s001.docx]

**Appendix S1: Mplus commands used for analyses**

**BSEM for HRQoL of TB patients**

Data: File is qol.dat ;

Names are pw1 pw2 pw3 pw4 pw5 pw6 pw7 mw1 mw2 mw3

mw4 mw5 mw6 mw7 sw1 sw2 sw3 sw4 sex age edu occu income

smoke drinking subsabus tqol tpqol ;

Missing are . ;

Use variables are pw1 pw2 pw3 pw4 pw5 pw6 pw7

mw1 mw2 mw3 mw4 mw5 mw6 mw7 sw1 sw2 sw3 sw4

age occu smoke drinking subsabus tpqol;

categorical are pw1-pw7 mw1-mw7 sw1-sw4 smoke drinking subsabus;

DEFINE:

if pw3<4 then pw3=4;

if pw4<2 then pw4=2;

if pw7<2 then pw7=2;

ANALYSIS:

processors is 8;

estimator is bayes;

biter is 250000;

MODEL:

pw by pw1 pw2 pw3 pw4 pw5 pw6 pw7;

mw by mw1 mw2 mw3 mw4 mw5 mw6 mw7;

sw by sw1 sw2 sw3 sw4;

habits by smoke drinking subsabus;

tpqol on pw mw sw habits;

pw mw sw habits tpqol on age occu ;

pw mw sw habits with pw mw sw habits;

OUTPUT: stdyx tech1 tech4 tech8 sampstat;

PLOT: type = plot2;
